# Design, Experimental Characterization and Finite Element Validation of Melt Electrowritten PCL/Hydrogel Composites for Pelvic Floor Tissue Repair

**DOI:** 10.3390/polym18151856

**Published:** 2026-07-29

**Authors:** Ana Telma Silva, Nuno Miguel Ferreira, Avener Santos, Ana Colette Maurício, Nuno Alves, Maria Elisabete Silva

**Affiliations:** 1 Faculty of Engineering (FEUP), University of Porto, Rua Dr. Roberto Frias s/n, 4200-465 Porto, Portugal; tcsilva@inegi.up.pt (A.T.S.); nmferreira@inegi.up.pt (N.M.F.); 2 Associated Laboratory for Energy, Transports and Aeronautics (LAETA), Institute of Science and Innovation in Mechanical and Industrial Engineering (INEGI), R. Dr. Roberto Frias 400, 4200-465 Porto, Portugal; 3 Biomaterials Laboratory (Poli-Bio), Institute of Chemistry, Federal University of Rio Grande do Sul, Av. Bento Gonçalves, 9500, Porto Alegre 91501-970, Brazil; eng.avenersantos@gmail.com; 4 Departamento de Clínicas Veterinárias, Instituto de Ciências Biomédicas de Abel Salazar (ICBAS), Universidade do Porto, Rua de Jorge Viterbo Ferreira, n° 228, 4050-313 Porto, Portugal; acmauricio@icbas.up.pt; 5 Associated Laboratory for Green Chemistry (LAQV) of the Network of Chemistry and Technology (REQUIMTE), Instituto de Ciências Biomédicas de Abel Salazar (ICBAS), Universidade do Porto, Rua de Jorge Viterbo Ferreira, 228, 4050-313 Porto, Portugal; 6 Centre for Rapid and Sustainable Product Development (CDRSP), Polytechnic Institute of Leiria (IPL), 2430-028 Marinha Grande, Portugal; nuno.alves@ipleiria.pt

**Keywords:** pelvic organ prolapse, melt electrowriting, hydrogel, finite element analysis

## Abstract

Conventional synthetic meshes for pelvic organ prolapse (POP) frequently cause severe complications, such as tissue erosion, due to a profound mechanical mismatch with native tissue. This study proposes a novel biphasic composite scaffold combining a load-bearing melt electrowritten (MEW) polycaprolactone (PCL) framework with a compliant alginate–gelatin (Alg-Gel) hydrogel matrix. PCL meshes (1.5 and 2.0 mm pores) were infiltrated with varying Alg-Gel ratios (4:3 and 5:2) and structurally evaluated through mechanical testing, swelling/degradation assays in a simulated acidic vaginal environment (pH 4.3), and finite element analysis (FEA). Results demonstrated that the 1.5 mm PCL architecture provides a robust baseline to withstand physiological loads. Notably, the hydrogel matrix provides a viscoelastic damping effect that synergistically improves the overall mechanical stability of the composite. Furthermore, FEA accurately predicted the non-linear macroscopic response of the composite constructs. Modulating the Alg-Gel ratio also enabled precise tuning of swelling capacity (up to 1400%) and degradation kinetics. Ultimately, this biomimetic system offers a highly adaptable, tissue-like protective cushion with enhanced dynamic stability, presenting a versatile platform for future in vivovalidation.

## 1. Introduction

Dynamic soft tissues are fundamental to human biomechanics, where they are uniquely evolved to withstand continuous, complex, and repetitive cyclic loading without compromising their structural integrity or flexibility [1]. The successful repair of defects within these highly active environments remains one of the most demanding challenges in modern biomedical engineering [2]. This challenge is particularly critical in the treatment of Pelvic Organ Prolapse (POP), where the pelvic floor must endure rigorous mechanical demands driven by constant variations in intra-abdominal pressure [3,4,5]. Traditional surgical interventions for pelvic floor reconstruction frequently rely on the implantation of permanent, rigid synthetic meshes, most commonly composed of polypropylene [6]. However, the profound elastic mismatch between these stiff synthetic materials and the compliant native tissue inevitably disrupts the natural biomechanical environment [7]. This mechanical incompatibility often leads to stress shielding, chronic inflammation, fibrosis, and, in severe clinical scenarios, debilitating adverse events such as tissue erosion [8,9].

To overcome these limitations, soft tissue engineering has increasingly shifted toward biomimetic composite scaffolds that replicate the natural biphasic architecture of native tissues [10]. The integration of synthetic and natural polymers is a well-established strategy that balances rigorous mechanical demands with biological compatibility [11,12]. Synthetic polymers, such as polycaprolactone (PCL), offer favorable mechanical properties, a slow degradation rate, and excellent printability, particularly when processed through advanced high-resolution techniques like melt electrowriting (MEW) [13,14,15,16]. However, while PCL provides the necessary macroscopic structural backbone, its inherent hydrophobicity and limited bioactivity restrict optimal cellular integration [17].

A highly effective strategy to bridge this mechanical–biological divide is the encapsulation of the rigid PCL architecture within a natural hydrogel matrix, which mimics the highly hydrated and compliant ground substance of the native extracellular matrix (ECM) [18]. Among natural biomaterials, blends of alginate (Alg) and gelatin (Gel) have emerged as highly synergistic matrices [19,20]. Alginate, a natural polysaccharide, functions as the primary structural anchor of the hydrogel. Driven by its rapid ionic crosslinking capabilities, alginate forms a stable three-dimensional network that dictates the scaffold’s density, fluid retention capacity, and long-term physical resilience in dynamic environments [21,22]. Conversely, gelatin is a protein derived from the hydrolysis of native collagen [23]. While alginate provides robust structural stability, it inherently lacks mammalian cell-adhesive motifs [24]. Gelatin bridges this biological gap by introducing essential biochemical cues, such as arginine–glycine–aspartic acid (RGD) peptide sequences, which are vital to enhance tissue adhesion, cell spreading, and overall cell–scaffold interactions [25,26,27]. Consequently, blending these two distinct biomaterials allows for the fine-tuning of the hydrogel’s physicochemical properties. In high-demand clinical applications like pelvic floor repair, this specific material hierarchy ensures that the structural stability governed by alginate remains the dominant driving force, complemented by the bioactivity of gelatin to provide a fully protective and biomimetic viscoelastic cushion [20,28]. Recent studies on MEW-reinforced hydrogels emphasize that the mechanical performance of these composites is highly dependent on the architecture of the polymeric framework, with parameters such as pore size and geometry playing a critical role in tailoring the load-bearing capacity of the scaffolds [29].

While experimental mechanical characterization is essential to evaluate the baseline properties of these composite scaffolds, empirical testing alone is often insufficient to fully capture the complex stress distributions and deformation patterns occurring at the microstructural level. Consequently, computational modeling, particularly Finite Element Analysis (FEA), has become a critical tool in modern biomaterial design [30]. In silico models allow for the simulation of the non-linear, hyperelastic behavior of soft matrices and their precise mechanical interaction with rigid structural frameworks [31]. By computationally replicating these multiaxial load transfer mechanisms, researchers can predict potential areas of stress concentration or interfacial failure under physiological-like conditions [32]. Integrating computational simulations with empirical data not only accelerates the optimization of scaffold geometries without the need for exhaustive experimental iterations but also provides a highly robust validation of the implant’s biomechanical compatibility for dynamic environments like the pelvic floor.

Building upon this rationale, the primary objective of this study was to design, fabricate, and characterize a biomimetic composite scaffold for pelvic floor tissue repair consisting of a MEW PCL framework infiltrated with an Alg-Gel hydrogel. Specifically, this work aimed to: (1) evaluate the influence of PCL pore architecture on the tensile behaviour of the scaffold; (2) validate a finite element model by comparison with the experimental tensile response; and (3) characterize the cyclic mechanical behaviour, compressive response, and swelling properties of different Alg-Gel formulations under simulated vaginal conditions. Ultimately, this study seeks to demonstrate the potential of this biphasic scaffold as a mechanically stable and biomimetic platform for future regenerative strategies in pelvic floor repair.

## 2. Materials and Methods

### 2.1. Materials

#### 2.1.1. Polycaprolactone (PCL)

The scaffold framework was fabricated using technical-grade PCL filament (Facilan PCL 100, 3D4Makers, Haarlem, The Netherlands, diameter 1.75 mm). This FDA-approved, hydrophobic, and semi-crystalline polymer was selected for its favorable mechanical properties and suitability for MEW [15,16]. Its physical characteristics include a density of 1.1 g/cm^3^, a melting range of 58 °C to 60 °C, a decomposition temperature of 200 °C, and a melt flow index between 5.2 and 11.3 g/10 min [30,33].

#### 2.1.2. Alginate

Sodium alginate (purity 99.8%, MW = 222.00 g/mol, MP Biomedicals, Porto, Portugal) was selected as the hydrogel precursor due to its excellent biocompatibility, predictable mechanical behavior, and rapid ionic crosslinking capabilities [31].

#### 2.1.3. Gelatin

Commercial gelatin from bovine skin (Type B, gel strength ∼225 g Bloom) was obtained from Sigma-Aldrich (Merck, Brazil). It was utilized to incorporate essential biological cues, such as RGD peptide sequences, into the hydrogel matrix to facilitate future cell–scaffold interactions.

#### 2.1.4. Crosslinking and Solvent Agents

Calcium chloride dihydrate ($CaCl_{2}\cdot 2H_{2}O$) with a molar mass of 147.02 g/mol was used as the ionic crosslinking agent to facilitate the formation of the alginate “egg-box” network. Phosphate-buffered saline (PBS, pH 7.4) was utilized as the solvent for all preparations. Because this study focuses exclusively on the structural and mechanical behavior of the composite without the incorporation of live cells, PBS was chosen as an optimal and simplified solvent over complex cell culture media. Furthermore, the use of PBS ensures a physiologically relevant ionic environment, which is critical for maintaining stable alginate mechanics and representative swelling behavior.

All chemical reagents and polymers were used as received without further purification.

### 2.2. Fabrication Processes

**Figure 1:**
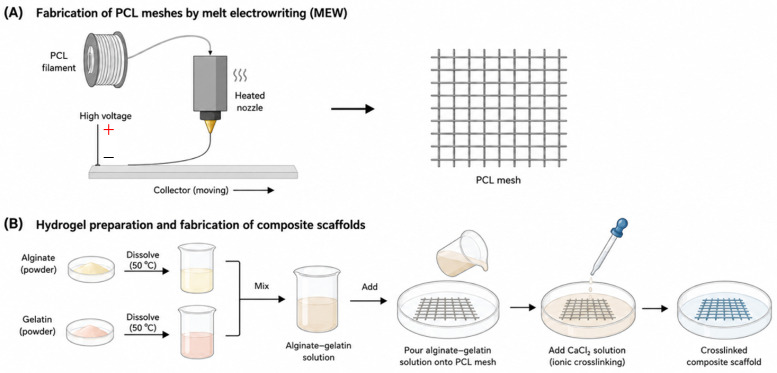
Schematic representation of the fabrication process of the biphasic composite scaffolds. (**A**) MEW process for the production of the PCL framework, highlighting the high-voltage electrical field and the moving collector system. (**B**) Preparation of the alginate–gelatin hydrogel and subsequent fabrication of the composite scaffold via infiltration of the PCL mesh.

The fabrication process of the biphasic composite scaffolds is schematically illustrated in Figure 1. This workflow encompasses two main stages: the production of the PCL framework via Melt Electrowriting (MEW) and the subsequent assembly of the PCL–hydrogel composite.

#### 2.2.1. Melt Electrowriting of PCL Meshes

PCL meshes were fabricated using a custom-built MEW system developed in-house. To ensure consistent print quality, the manufacturing environment was strictly controlled (21.5 °C, 49% relative humidity). Geometric designs were programmed using FullControl software (v3), an open-source software that enables the generation of function-controlled print paths [34]. This software allowed for the optimization of the print paths by calculating the necessary filament diameter for each segment, ensuring high geometric accuracy. The scaffolds were printed with a quadratic architecture consisting of two orthogonal fiber layers with a fiber diameter of 480 µm. To evaluate the effect of pore dimensions on the final composite, two groups were printed with fixed dimensions of 10 mm × 40 mm, featuring pore sizes of either 1.50 mm or 2.00 mm. These specific pore sizes were chosen to encourage optimal cell growth and assist in POP repair [35].

#### 2.2.2. Preparation of Alginate–Gelatin Hydrogels

The hydrogel matrices were prepared at a constant total polymer concentration of 5% (*w*/*v*) in PBS, with variations in the alginate-to-gelatin mass ratio. Following established strategies [20], alginate was maintained as the dominant phase to ensure robust crosslinking, while gelatin was added at lower proportions to provide cell-adhesive RGD motifs. Therefore, the 4:3 and 5:2 ratios were selected to comparatively evaluate how varying the gelatin content influences the physical and mechanical properties of the composite. The formulations were prepared as follows:

- **Alg-Gel 5:2**: 0.5 g of sodium alginate and 0.2 g of gelatin dissolved in 14 mL of PBS.
- **Alg-Gel 4:3**: 0.4 g of sodium alginate and 0.3 g of gelatin dissolved in 14 mL of PBS.
- **Control**: Pure alginate hydrogel (5% *w*/*v*).

All mixtures were subjected to continuous magnetic stirring at 50 °C until achieving homogeneous, highly viscous, and bubble-free solutions.

#### 2.2.3. Mesh–Hydrogel Composite Assembly

The MEW-printed PCL meshes were carefully positioned in flat molds and completely infiltrated with the uncrosslinked hydrogel precursor solutions. Upon total infiltration into the 1.5 mm and 2.0 mm macropores, the $CaCl_{2}$ solution was applied to initiate the ionic gelation process. The diffusion of $Ca^{2+}$ ions converted the liquid precursor into a stable, viscoelastic hydrogel interlocked within the PCL framework. Following a 30 min curing period at room temperature, the composites were rinsed with ethanol to reduce the hydrophobicity of the PCL framework, thereby enhancing its wettability by the hydrogel precursor. Subsequently, samples were washed with physiological saline to remove residual crosslinking agents, effectively terminating the ionic crosslinking reaction and ensuring sample stability.

### 2.3. Swelling Behavior Assessment

To evaluate the fluid uptake capacity and structural stability of the composite hydrogels in a relevant physiological scenario, swelling tests were conducted. To accurately mimic the acidic conditions of the vaginal microenvironment, PBS was adjusted to a pH of 4.3 using dilute hydrochloric acid (HCl) [36,37]. The pre-weighed samples were immersed in the acidified PBS solution and maintained at 37 °C. At predetermined time intervals (15, 30, 45, and 60 min, as well as 1 and 3 days), the samples were carefully removed from the solution. Excess surface fluid was gently blotted using filter paper, and the samples were immediately weighed to determine the wet mass. The swelling ratio was subsequently calculated based on the percentage increase in mass relative to the initial state, allowing for the assessment of the hydrogels’ structural integrity and absorption kinetics over a 72 h period in the simulated vaginal environment [38].

### 2.4. Experimental Characterization

To ensure the reliability and reproducibility of the data, all mechanical evaluations—including tensile, cyclic, and compressive tests—were performed in triplicate (*n* = 3).

#### 2.4.1. Mechanical Testing

**Figure 2:**
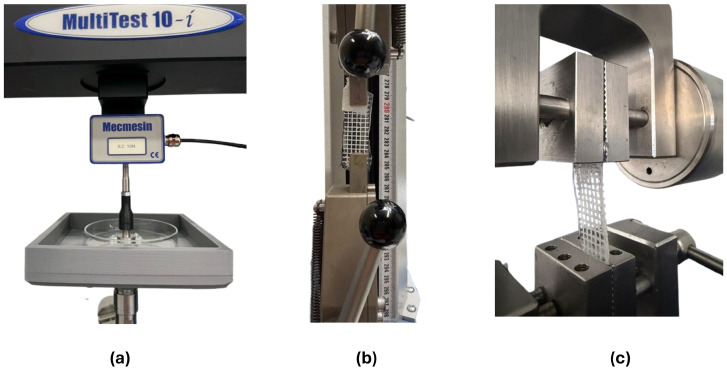
Experimental configurations employed for the mechanical characterization of the meshes and hydrogels. (**a**) Unconfined compression testing setup utilizing a 10 N load cell for the evaluation of the isolated hydrogels. (**b**) Uniaxial tensile testing equipped with standard mechanical grips and a 100 N load cell. (**c**) Cyclic fatigue testing configuration utilizing pneumatic grips to ensure uniform pressure and prevent sample slippage during repeated loading.

All mechanical evaluations were performed using a universal testing machine (Mecmesin, West Sussex, UK) at a constant crosshead speed of 1 mm/min, conducted at room temperature. Different load cells and grip configurations were utilized depending on the specific testing modality (Figure 2).

##### Compression Testing

To characterize the intrinsic viscoelastic properties of the hydrogel matrices independently of the PCL framework, unconfined compression tests were conducted. Compression was applied using a flat-ended cylindrical plunger with diameter of 18.75 mm, equipped with a 10 N load cell. A preload of 100 mN was applied to all samples to ensure uniform contact before testing.

##### Uniaxial Tensile Testing

Tensile properties were initially assessed for the bare MEW PCL meshes to evaluate the influence of pore size (1.5 mm vs. 2.0 mm). Rectangular specimens with dimensions of 40 mm × 10 mm were secured using standard mechanical grips, and force–displacement data was recorded using a 100 N load cell. Following the determination of the optimal mechanical behavior in the 1.5 mm pore meshes, this configuration was selected for all subsequent composite fabrication and testing.

##### Cyclic Testing

To simulate in vivo physiological conditions, load-controlled cyclic tensile tests were performed on the composite meshes using a 500 N load cell and pneumatic grips to prevent sample slippage. The maximum load was established at 80% of the ultimate tensile force (approximately 16 N), with a minimum force of 3 N established during the unloading phase. Samples were subjected to 100 consecutive load–unload cycles to evaluate hysteresis, cyclic mechanical stability, and accumulated plastic deformation.

### 2.5. Computational Models

#### 2.5.1. Mechanical Characterization

To complement the experimental characterization, a finite element analysis was performed to simulate the mechanical response of the PCL reinforcing mesh and the hydrogel. The reinforcing mesh was assumed to be made of PCL and was modeled as an isotropic elastic–plastic material.

Properties used in the simulations were directly obtained from our own experimental mechanical testing of the PCL material. Specifically, the material was assigned a density of 1140 kg/m^3^, a Young’s modulus of 320 MPa, and a Poisson’s ratio of 0.30. Plastic deformation was defined using the true stress–plastic strain data summarized in Table 1. These material properties were implemented in Abaqus to describe the mechanical response of the reinforcing mesh during loading.

The alginate hydrogel was modeled as a nearly incompressible hyperelastic material to capture its nonlinear mechanical response under large deformations. Its constitutive behaviour was described using the Yeoh hyperelastic model [31], whose material parameters were identified by fitting the experimental uniaxial compression data, as described in previous Section 2.4.1. The strain energy density function of the Yeoh model is expressed as

(1) $$W=c_{1}\left({\bar{I}}_{1}-3\right)+c_{2}{\left({\bar{I}}_{1}-3\right)}^{2}+c_{3}{\left({\bar{I}}_{1}-3\right)}^{3},$$

where $c_{1}$, $c_{2}$, and $c_{3}$ are the material constants, and ${\bar{I}}_{1}$ is the first deviatoric invariant of the modified right Cauchy–Green deformation tensor. The resulting coefficients used in the finite element simulations are listed in Table 2.

#### 2.5.2. Geometric Modeling

**Figure 3:**
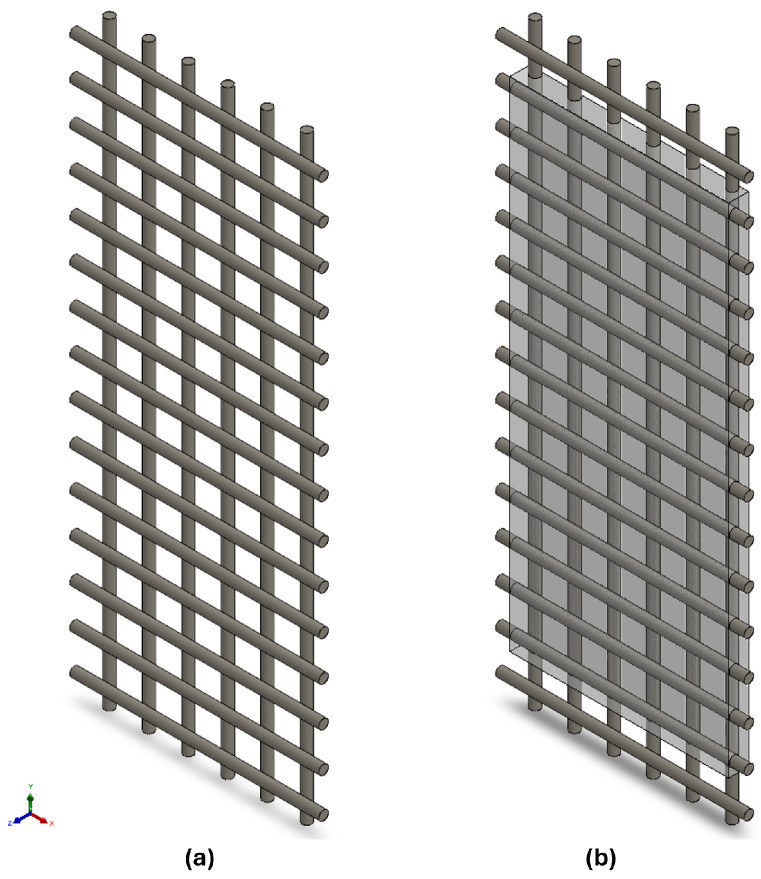
Finite element model showing (**a**) the reinforcing mesh and (**b**) the assembled hydrogel–mesh composite used in the simulations.

A three-dimensional finite element model was developed in Abaqus to reproduce the experimental configuration. The model consisted of a reinforcing mesh embedded within an alginate hydrogel matrix, as illustrated in Figure 3. The reinforcing mesh measured 30 mm in height, 12.5 mm in width, and 0.96 mm in thickness, corresponding to two overlapping filaments, each with a diameter of 0.48 mm. The hydrogel matrix measured 25 mm in height, 11 mm in width, and 1 mm in thickness. The hydrogel and reinforcing mesh were modeled as separate parts and assembled to reproduce the fabricated specimen.

The hydrogel was discretized using four-node linear tetrahedral hybrid elements (C3D4H), which are suitable for nearly incompressible hyperelastic materials. The reinforcing mesh was discretized using four-node linear tetrahedral elements (C3D4), as only linear elastic behaviour was considered for this component.

A uniform global element size of 0.5 mm was adopted for all models, validated by a mesh convergence study evaluating sizes from 0.25 to 1.00 mm. This ensured minimal variation in the predicted maximum force, providing an optimal balance between computational efficiency and accuracy [39]. General contact was defined between all interacting surfaces to ensure mechanical interaction between the hydrogel matrix and the reinforcing mesh throughout the loading process.

### 2.6. Statistical Analysis

Data from triplicate experiments (*n* = 3) are expressed as mean ± standard deviation (SD). Statistical significance (*p* < 0.05) was determined using one-way ANOVA followed by Tukey’s post hoc test, after verifying normality (Shapiro–Wilk) and variance homogeneity (Levene’s test) using IBM SPSS Statistics (version 31, IBM Corp., Armonk, NY, USA).

## 3. Results

To accurately reflect the non-linear and viscoelastic material behavior, continuous graphical representations (such as stress–strain and load–displacement plots) depict representative curves for each experimental group.

### 3.1. Swelling Behavior in Simulated Vaginal Environment

**Figure 4:**
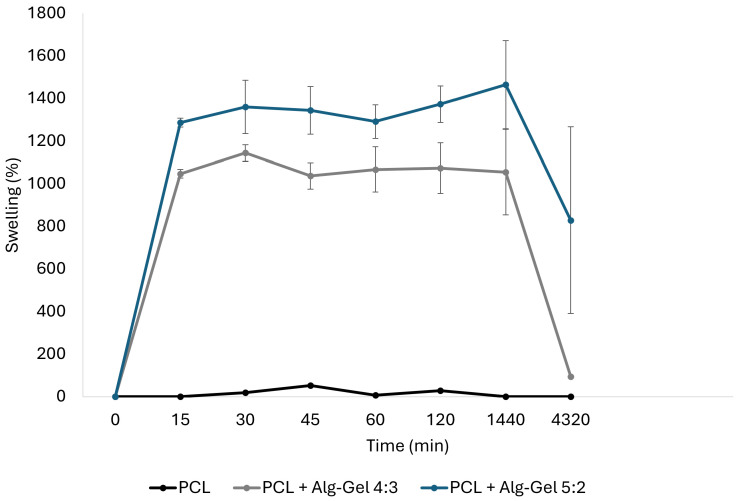
Swelling kinetics of the bare PCL mesh and PCL–hydrogel composite scaffolds (Alg-Gel 4:3 and Alg-Gel 5:2) over a 72 h period (4320 min) in a simulated vaginal environment (pH 4.3). The composite scaffolds demonstrate rapid initial fluid uptake and stable retention for 24 h, followed by formulation-dependent degradation profiles at 72 h. Data are presented as mean ± standard deviation (n = 3).

The swelling kinetics of the MEW PCL meshes and PCL–hydrogel composites in a simulated vaginal environment (pH 4.3) are presented in Figure 4. As expected, the bare PCL meshes exhibited negligible swelling due to the hydrophobic nature of the polyester framework. In contrast, the composite scaffolds displayed rapid fluid uptake within the first 15 min, reaching a steady-state equilibrium shortly thereafter. The PCL + Alg-Gel 5:2 formulation demonstrated the highest swelling capacity, plateauing at approximately 1400 ± 206%, while the PCL + Alg-Gel 4:3 group stabilized near 1050 ± 210%. This enhanced water uptake is attributed to the higher alginate content in the 5:2 formulation, which increases the hydrophilic character of the hydrogel network.

Remarkably, both composite systems maintained their structural integrity and peak swelling capacity throughout a 24 h period (1440 min). However, a significant decrease was observed at 4320 min (72 h), suggesting the onset of hydrogel degradation or physical dissolution in the acidic medium. Notably, the Alg-Gel 4:3 composite experienced a severe loss of its swelling capacity (dropping to approximately 100 ± 3%), whereas the 5:2 formulation retained a substantial swelling ratio of ∼800 ± 408%. The increased variability observed at this later time point is attributed to the progressive macroscopic degradation and heterogeneous partial mass loss of the hydrogel in the acidic environment. This indicates that the higher alginate ratio provides superior long-term structural stability against degradation. Overall, these findings demonstrate the high fluid uptake capacity of the hydrogel formulations, highlighting their potential for future incorporation and delivery of bioactive molecules.

### 3.2. Compressive Response of the Hydrogel Phase

**Figure 5:**
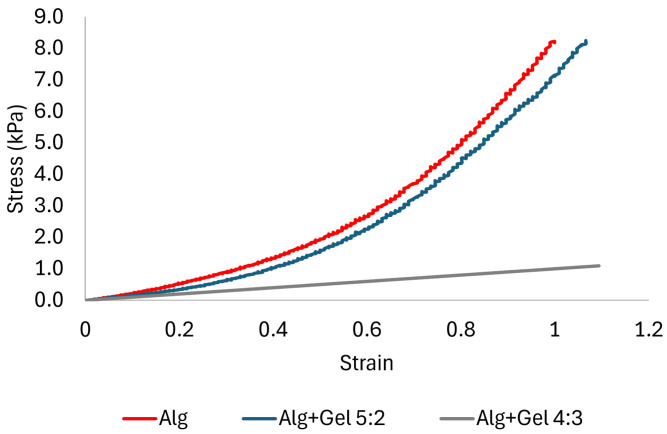
Representative compressive stress–strain curves of the isolated hydrogel formulations (Alg, Alg+Gel 5:2, and Alg+Gel 4:3).

The compressive properties of the isolated hydrogels were evaluated to characterize their inherent stiffness (Figure 5). All formulations displayed a non-linear, strain-dependent behavior characteristic of soft, hyperelastic materials. The Alg group exhibited the highest compressive stress values, indicating a higher stiffness compared to the Alg-Gel hybrid groups. Among the composite formulations, the Alg-Gel 5:2 group demonstrated a superior mechanical response compared to the Alg-Gel 4:3 group, reaching significantly higher stress levels at equivalent strain percentages. These results demonstrate that the mechanical properties of the hydrogel can be tailored by modifying the alg-to-gel ratio, providing a strategy to tailor the overall mechanical behaviour of the composite scaffold.

### 3.3. Mechanical Performance of the Composite Scaffolds

#### 3.3.1. Tensile Properties and Statistical Analysis

**Figure 6:**
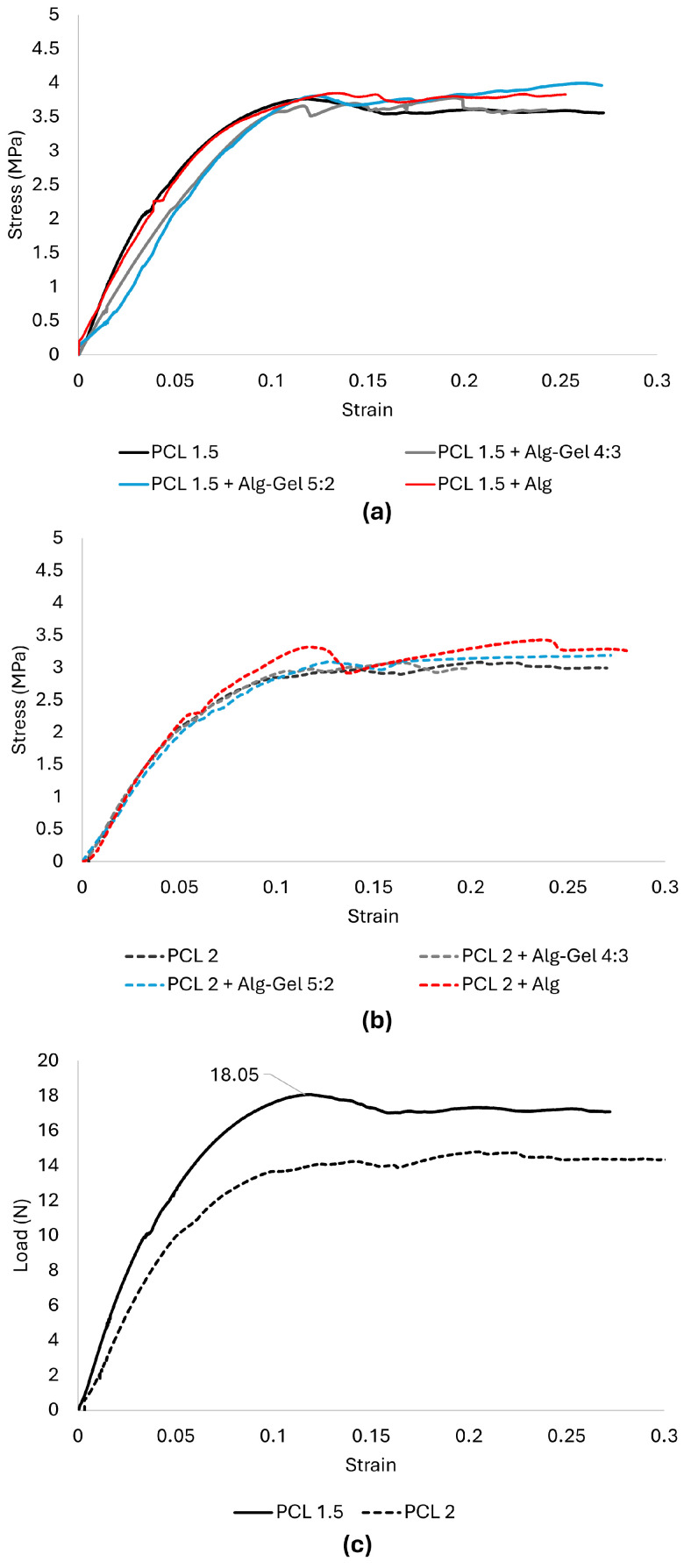
Tensile mechanical properties of the fabricated scaffolds. The stress–strain curves demonstrate the effect of hydrogel incorporation (Alg-Gel 4:3, Alg-Gel 5:2, and Alg) on the mechanical response of (**a**) PCL 1.5 and (**b**) PCL 2 polymeric base matrices. (**c**) Representative load–strain curves of the neat PCL 1.5 and PCL 2 meshes, highlighting the maximum tensile load (18.05 N for PCL 1.5).

The tensile properties of the PCL meshes and the PCL–hydrogel composites are summarized in Figure 6.

The bare PCL meshes with 1.5 mm pore size exhibited superior mechanical performance compared to the 2.0 mm configuration, achieving an average maximum force of approximately 18 N. As illustrated in Figure 6a,b, the stress–strain profiles demonstrate that the inclusion of the hydrogel phase maintains the structural integrity of the PCL framework. The similarity in the stress–strain curves suggests that the hydrogel matrix effectively integrates with the PCL fibers without inducing stress concentrations, promoting a uniform load distribution across the scaffold structure. Based on its superior tensile performance, the 1.5 mm pore size configuration was selected for the fabrication and subsequent evaluation of the composite scaffolds.

**Figure 7:**
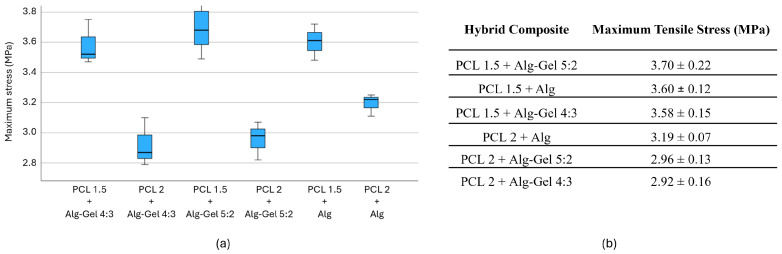
Quantitative comparison of the maximum tensile stress obtained for the different scaffold configurations. (**a**) Boxplots showing the distribution of the experimental maximum tensile stress values. (**b**) Mean ± SD of the maximum tensile stress for each scaffold configuration (n = 3). Statistical analysis was performed using one-way ANOVA followed by Tukey’s HSD post hoc test (*p* < 0.05).

To quantitatively compare the tensile performance of the different scaffold configurations, the maximum tensile stress values were statistically analysed (Figure 7). One-way ANOVA revealed significant differences among the six scaffold configurations (F(5,12) = 16.269, *p* < 0.001). Tukey’s HSD post hoc analysis showed that scaffolds with a 1.5 mm pore size exhibited significantly higher maximum tensile stress than their corresponding 2.0 mm pore size counterparts. In contrast, no significant differences were observed among the different hydrogel formulations within the same pore size in most pairwise comparisons.

Although the one-way ANOVA demonstrated significant differences among the scaffold configurations, Tukey’s HSD post hoc analysis was performed to identify the specific scaffold pairs exhibiting statistically significant differences. The significant pairwise comparisons are summarized in Table 3.

#### 3.3.2. Cyclic Resistance

**Figure 8:**
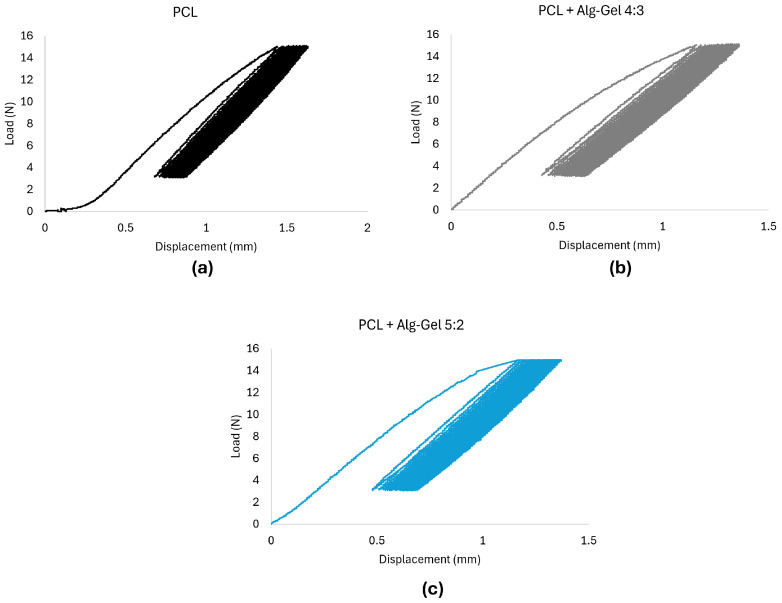
Cyclic load–displacement curves of the fabricated scaffolds. The tests were performed up to a maximum load of 14.44 N (representing 80% of the maximum static load) for (**a**) the neat PCL mesh, (**b**) PCL + Alg-Gel 4:3, and (**c**) PCL + Alg-Gel 5:2. The hysteresis loops highlight the viscoelastic nature of the materials, with hydrogel-loaded groups demonstrating reduced maximum displacement and enhanced structural stability under repetitive cyclic loading.

To evaluate the dynamic mechanical stability of the scaffolds under repetitive loading, cyclic tensile tests were performed. Based on the static tensile results, a threshold corresponding to 80% of the maximum load of the neat PCL mesh (14.44 N) was applied. As observed in Figure 8, all groups exhibited typical viscoelastic behavior characterized by distinct hysteresis loops during the loading–unloading cycles.

A pronounced initial plastic deformation occurred in the first cycle for all samples, representing the alignment and structural accommodation of the polymer network. However, the incorporation of the hydrogel within the PCL matrix significantly influenced the cyclic response. The neat PCL scaffolds (Figure 8a) reached a maximum displacement of ∼1.6 mm. In contrast, the composite scaffolds containing Alg-Gel 4:3 (Figure 8b) and Alg-Gel 5:2 (Figure 8c) demonstrated enhanced structural stability, reducing the maximum displacement to approximately 1.4 mm under the same load. Furthermore, the hydrogel-infiltrated meshes exhibited lower accumulated permanent deformation than the neat PCL scaffold, indicating an improved resistance to irreversible stretching during repeated physiological loading.

### 3.4. Finite Element Model Validation

**Figure 9:**
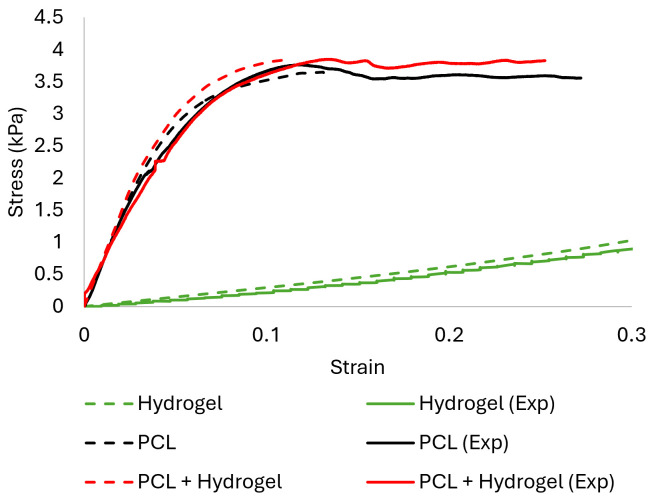
Validation of the computational model. The tensile stress–strain curves show an excellent correlation between the experimental data (solid lines) and the numerical Finite Element Analysis predictions (dashed lines) for the isolated hydrogel, neat PCL, and the composite scaffold.

Finite element simulations were performed to reproduce the experimental tensile tests and validate the computational model. Figure 9 presents the comparative tensile stress–strain curves between the experimental data (solid lines) and the computational simulations (dashed lines).

The numerical model demonstrated excellent agreement with the experimental results across all three evaluated groups (isolated hydrogel, bare PCL, and PCL–hydrogel composite), accurately capturing the non-linear, hyperelastic behavior of the constructs. Quantitatively, the FEA predictions for the maximum stress deviated by less than 10% from the experimental values, demonstrating good agreement between the numerical model and the experimental measurements. The composite scaffold (PCL + hydrogel) exhibited a slightly higher tensile stiffness than the bare PCL scaffold, in agreement with both the experimental and computational results.

**Figure 10:**
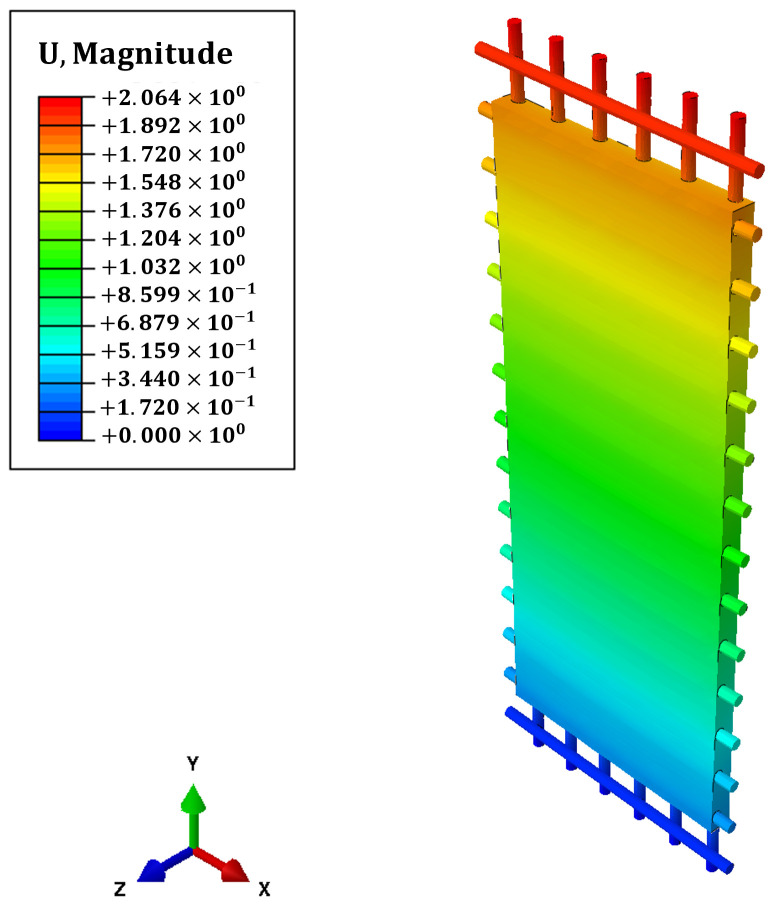
Representative finite element contour plot showing the displacement magnitude (U) of the composite scaffold under tensile loading. The displacement field illustrates a smooth and continuous deformation throughout the scaffold.

The integration of the two phases can be visually assessed through the displacement field distribution obtained from the FEA (Figure 10). The contour plot reveals a uniform and progressive displacement gradient from the fixed base to the loaded surface. This homogeneous deformation pattern indicates compatible deformation between the rigid PCL framework and the compliant hydrogel matrix under tensile loading. The robust validation of this computational model provides a reliable tool for future geometric optimizations of these composite scaffolds without the immediate need for exhaustive experimental iterations.

## 4. Discussion

The present study successfully developed and characterized a novel composite scaffold comprising a MEW PCL framework integrated with an Alg-Gel hydrogel matrix. Designed specifically for pelvic floor repair, this biphasic system aims to overcome the well-documented complications of conventional polypropylene meshes, such as mechanical mismatch and severe tissue erosion [7,8]. By combining the load-bearing capacity of a rigid polyester with the compliance and hydration properties of a hyperelastic hydrogel, the resulting composite provides a tunable, viscoelastic response designed to better accommodate the dynamic biomechanical environment of the pelvic floor [10,18].

The mechanical performance of the scaffolds demonstrated that the macroscopic structural integrity is primarily governed by the PCL architecture. The 1.5 mm pore size configuration exhibited superior tensile strength compared to the 2.0 mm variant, providing a robust baseline capable of withstanding physiological intra-abdominal pressures. This strong dependence on pore size closely aligns with the findings of Trucco et al. [29], who demonstrated that reducing the pore size of MEW frameworks significantly enhances the tensile modulus and overall load-bearing capacity of hydrogel composites. This behavior is also in agreement with other recent studies, which highlight the critical role of MEW architectures in defining the ultimate mechanical resilience of prolapse meshes [15,33]. Importantly, the integration of the hydrogel did not compromise this tensile strength but rather fostered a synergistic mechanical response, a biphasic reinforcement phenomenon similarly observed by Zhang et al. in other soft tissue engineering platforms [11]. Our computational FEA corroborated these empirical findings, revealing a uniform displacement gradient and accurately predicting the non-linear macroscopic response of the composite constructs. Rather than extracting single linear moduli or toughness values, this study prioritized full non-linear deformation curves to enable direct future comparisons with native pelvic tissues. As previously demonstrated by Knight et al. [32], predicting and ensuring this macroscopic structural stability is critical to prevent localized stress concentrations that typically lead to progressive plastic deformation, pore collapse, and subsequent implant failure.

The native pelvic floor is continuously subjected to dynamic loading regimens—such as coughing, laughing, or lifting—making cyclic mechanical stability a paramount design criterion for POP implants [1,5]. Under cyclic tensile testing, the neat PCL meshes exhibited a pronounced initial plastic deformation, a well-documented drawback frequently observed in purely synthetic devices. However, the incorporation of the Alg-Gel matrix significantly enhanced the dynamic structural stability of the constructs. In this biphasic system, the hydrogel provides a viscoelastic damping effect. By interlocking with the PCL network, it appears to restrict the excessive mobility of the fibers and notably reduces both the maximum displacement and the accumulated permanent strain. This energy dissipation mechanism aligns with the principles of load-bearing tissue regeneration described by Chen et al. [2], who demonstrated that compliant biomimetic matrices are essential to efficiently distribute repetitive cyclic stresses. Consequently, this enhanced dimensional stability ensures that the implant can sustain prolonged physiological demands without undergoing irreversible elongation, a biomechanical failure widely identified as a primary clinical cause of prolapse recurrence [3,8]. The selected cyclic loading protocol (100 cycles) is consistent with methodologies previously reported for the mechanical evaluation of pelvic floor repair devices. Notably, Pinheiro et al. [40] employed an analogous 100-cycle loading protocol to evaluate the mechanical resilience of PCL-based structures for pelvic organ prolapse rehabilitation, supporting the suitability of this approach for assessing the immediate mechanical stability of PCL-based constructs. Furthermore, the selected cyclic load corresponds to 80% of the ultimate tensile force of the neat PCL scaffold, a commonly adopted strategy for evaluating elastic recovery and progressive plastic deformation while avoiding premature failure. Nevertheless, it is important to note that this protocol evaluates acute cyclic stability and immediate viscoelastic recovery, rather than the long-term fatigue behaviour expected under physiological conditions.

Beyond the structural framework, the composition of the hydrogel phase plays a critical role in defining the scaffold’s overall behavior, particularly in a simulated vaginal environment (pH 4.3). The evaluation of different Alg-Gel ratios demonstrated the highly tunable nature of the composite. While the Alg-Gel 5:2 formulation exhibited higher compressive stiffness and fluid uptake compared to the 4:3 ratio, both variations presented distinct and valuable degradation profiles. As extensively characterized by Di Giuseppe et al. [20] regarding the mechanical behavior of these blends, a higher alginate content yields a denser ionically crosslinked network. In our study, this translated into formulation-dependent swelling capacities and varying degradation kinetics. The 4:3 formulation exhibited a faster structural breakdown after 72 h in the acidic medium, whereas the 5:2 composite retained a more prolonged structural resilience. This ability to tailor the degradation rate and chemical resilience is crucial for designing hydrogel components adaptable to the harsh, naturally acidic environment of the vagina, a tunability under low pH that is highly consistent with the demanding criteria for vaginal delivery platforms highlighted in recent reviews on non-parenteral in situ gelling systems [41].

Clinically, the pronounced swelling and soft compressive properties of the hydrogel phase offer a protective, tissue-like cushion that shields the vaginal mucosa from the underlying rigid PCL fibers. This biomimetic interface provides a compelling strategy to mitigate the risk of tissue erosion and dyspareunia, which remain the primary causes of reoperation in conventional synthetic mesh surgeries [42].

Despite these promising mechanical, computational, and physicochemical findings, certain study limitations must be acknowledged. First, the in vitro swelling and degradation assays do not fully capture the complex enzymatic activity and multiaxial mechanical stress state present in vivo. Second, direct microstructural visualization via Scanning Electron Microscopy (SEM) was not performed to directly image the hydrogel–PCL interface, although matrix infiltration and interfacial coupling were indirectly supported by swelling kinetics and dynamic mechanical testing. Although Fourier-Transform Infrared (FTIR) analysis was not performed, the successful crosslinking of the alginate–gelatin network is supported by the established literature [28] and the observed structural and swelling stability. Furthermore, while the long-term degradation profile of the MEW PCL framework has been previously established by our group [43], long-term degradation profiling of the composite falls outside the scope of this study and will be addressed in future work, alongside detailed rheological characterization of the hydrogels. Finally, a key limitation of this work is the absence of direct in vitro biological validation (such as cytocompatibility, cell viability, and proliferation assays) for the finalized composite construct. While the individual components possess well-documented biological safety profiles in similar applications [44,45], the primary scope of this baseline study was strictly restricted to physical–mechanical characterization and numerical model validation. Consequently, comprehensive biological evaluations—including dynamic cell seeding, metabolic activity, extracellular matrix deposition, and subsequent in vitro animal studies—represent an essential next steps and will be the principal foci of upcoming investigations prior to translational application.

## 5. Conclusions

In conclusion, a biomimetic composite scaffold combining a MEW PCL framework with an Alg-Gel hydrogel matrix was successfully engineered for pelvic floor repair. The rigid PCL mesh provides the necessary mechanical baseline to withstand intra-abdominal pressures, while the hydrogel matrix provides a viscoelastic damping effect, significantly enhancing cyclic mechanical stability and reducing accumulated permanent deformation. Furthermore, modifying the hydrogel polymer ratio allowed for the precise tuning of the swelling capacities and degradation kinetics in a simulated acidic vaginal environment. By effectively bridging the mechanical mismatch and offering a highly adaptable, tissue-like protective interface, this biphasic system presents a versatile platform to mitigate conventional synthetic mesh complications, paving the way for future in vivo validation.

## Figures and Tables

**Table 1:** Mechanical properties of the PCL reinforcing mesh.

Property	Value	Unit
Density	1140	kg/m^3^
*E*	320	MPa
*ν*	0.30	–
	**True stress (MPa)**	**Plastic strain**
**Plastic properties**	9.5	0.000
	13.4	0.007
	15.6	0.017
	16.9	0.026
	17.3	0.040
	18.1	0.050
	18.7	0.070
	19.0	0.094

**Table 2:** Yeoh hyperelastic material parameters obtained from fitting the experimental compression test data of the alginate hydrogel.

*c*_1_ [MPa]	*c*_2_ [MPa]	*c*_3_ [MPa]
0.46374	0.34966	0.00453

**Table 3:** Statistically significant pairwise comparisons of the maximum tensile stress between scaffold configurations, as determined by Tukey’s HSD post hoc test following one-way ANOVA. Only statistically significant comparisons (*p* < 0.05) are reported.

Scaffold Configuration	Significantly Different from
PCL 1.5 + Alg-Gel 4:3	PCL 2 + Alg-Gel 4:3 (*p* = 0.002)
	PCL 2 + Alg-Gel 5:2 (*p* = 0.003)
PCL 1.5 + Alg-Gel 5:2	PCL 2 + Alg-Gel 4:3 (*p* < 0.001)
	PCL 2 + Alg-Gel 5:2 (*p* < 0.001)
	PCL 2 + Alg (*p* = 0.013)
PCL 1.5 + Alg	PCL 2 + Alg-Gel 4:3 (*p* = 0.001)
	PCL 2 + Alg-Gel 5:2 (*p* = 0.002)
	PCL 2 + Alg (*p* = 0.049)

## Data Availability

The original contributions presented in this study are included in the article. Further inquiries can be directed to the corresponding author.

## Abbreviations

The following abbreviations are used in this manuscript:

Alg	Alginate
ECM	Extracellular matrix
FDA	Food and Drug Administration
FEA	Finite Element Analysis
Gel	Gelatin
MEW	Melt Electrowriting
PCL	Polycaprolactone
POP	Pelvic Organ Prolapse
RGD	Arginine-glycine-aspartic acid
SD	Standard deviation
SEM	Scanning Electron Microscopy
